# A Comprehensive Analysis of the Prognostic, Immunological and Diagnostic Role of CCNF in Pan-cancer

**DOI:** 10.7150/jca.86597

**Published:** 2023-07-31

**Authors:** Xiaofeng Gao, Huitong Bu, Juanjuan Ge, Xuzheng Gao, Ying Wang, Zhenwang Zhang, Long Wang

**Affiliations:** 1 School of Basic Medical Sciences, Xianning Medical College, Hubei University of Science and Technology, Hubei University of Science and Technology, Xianning 437000, Hubei, PR China.; 2 Medicine Research Institute /Hubei Key Laboratory of Diabetes and Angiopathy, Xianning Medical College, Hubei University of Science and Technology, Xianning 437000, Hubei, PR China.; 3 College of Biology, Hunan University, Hunan, Changsha, 410012, PR China.; 4 School of Stomatology and Ophthalmology, Xianning Medical College, Hubei University of Science and Technology, Xianning 437000, Hubei, PR China.

**Keywords:** CCNF, Pan-Cancer, Diagnosis, Prognosis, Immunization, Biomarkers

## Abstract

**Background:** Cyclin F (CCNF) represents a pivotal constituent within the family of cell cycle proteins, which also belongs to the F-box protein family and acts as a critical regulatory factor in cell cycle transition. Its heightened expression has been consistently identified across various cancer types, including breast, pancreatic, and colorectal cancer. Nonetheless, a comprehensive exploration of CCNF's involvement in pan-cancer remains lacking.

**Methods:** This study collected transcriptomic data and clinical information from several databases, including The Cancer Genome Atlas (TCGA), Genotype-Tissue Expression (GTEx), and BioGPS detabase. Employing bioinformatics methods, we investigated the potential oncogenic role of CCNF, utilizing various databases such as cBioPortal, Human Protein Atlas (HPA), TIMER2, UALCAN, GEPIA, GSCALite, and CTD detabase. These analyses focused on exploring CCNF expression, prognosis, gene mutations, immune cell infiltration, DNA methylation levels, and targeted chemical drugs across different tumor types. Additionally, we obtained CCNF-related genes from GeneMANIA and GEPIA databases and conducted GO and KEGG enrichment analyses to gain deeper insights into the biological processes associated with CCNF. Furthermore, we validated the differential expression of CCNF in normal human breast cancer and breast cancer cell lines using experimental verification.

**Results:** CCNF exhibited upregulation in the majority of cancer types, demonstrating early diagnostic potential in 15 cancers and prognostic implications for adverse outcomes across numerous malignancies. Furthermore, CCNF was found to be linked with markers of the tumor immune microenvironment in various cancers. Additionally, CCNF expression influenced genetic alterations in pan-cancer. Enrichment analysis revealed that CCNF primarily participates in crucial biological pathways such as the cell cycle, p53 signaling pathway, and cellular senescence pathways. RT-qpcr and WB assays further confirmed that CCNF expression was higher in human cancer cell lines than in normal cell lines.

**Conclusion:** The underlying role and mechanism of CCNF in pan-cancer were elucidated through comprehensive bioinformatics analysis and experimental validation. CCNF holds promise as an invaluable early detection indicator and tumor biomarker, offering potential targets for tumor treatment and prevention.

## Introduction

Cancer constitutes a considerable threat to human life, and its morbidity and mortality are increasing yearly 1. With the development of medical technology, biologically targeted therapy and immunotherapy have become hot spots in tumor treatment 2, However, the inherent heterogeneity and recurrence of tumors present significant challenges in tumor treatment 3. Thus, there arises a critical need to discern novel biomarkers for tumor-related diagnosis, prognosis, and therapeutic interventions.

Cyclin F (CCNF), an essential member of the F-box and cell cycle protein family, was initially reported in 1994 4. CCNF serves as the substrate recognition subunit of Skp1-Cul1-F-box (SCF) E3 ubiquitin ligase complexes, exerting regulatory control over various cell cycle processes. This role encompasses the binding and/or ubiquitinating several cell cycle-associated protein substrates 5, including participation in centrosome replication regulation 6, maintenance of mitotic fidelity and genome stability 7, as well as DNA replication and repair 8. CCNF exhibits widespread expression in humans, nevertheless, the expression of CCNF varies across different tissues, with varying levels of CCNF expression 4. Higher expression levels are observed in skeletal muscle and brain in comparison to the heart and pancreas, while the lung, skin, bone marrow, and immune system exhibit the highest levels of expression 9. Furthermore, CCNF expression varies across cell cycles, exhibiting accumulation during the S phase, reaching its peak in the G2 phase, and subsequently decreasing during the M phase 4, 10. There is evidence that F-box proteins have emerged as critical regulators of cancer progression, exerting control over cell cycle-associated mediators that can either promote or inhibit cancer development 11, 12. Furthermore, the degradation of ribonucleotide reductase subunit M2 (RRM2) mediated by CCNF ensures the maintenance of balanced deoxyribonucleotide triphosphates (dNTPs) levels and controls the levels of centriolar coiled-coil protein 110 (CP110) through ubiquitin-mediated protein hydrolysis, thereby preventing mitotic aberrations caused by centrosome dysregulation 13, 14. Notably, recent studies have highlighted the upregulation of CCNF in ovarian cancer, with high CCNF expression correlating with unfavorable prognostic outcomes in ovarian cancer patients 15. Similarly, in breast cancer, CCNF overexpression has been associated with increased malignancy and a higher risk of metastatic recurrence, indicative of an adverse prognosis 16. Interestingly, Fu et al. reported contrasting findings, observing that elevated CCNF mRNA and protein expression were associated with improved prognosis in patients with hepatocellular carcinoma (HCC) 17. However, the exploration of CCNF's role has been confined to a limited number of tumor types, and its potential as an immunotherapeutic target in pan-cancer settings has yet to be investigated.

With the development of public databases and bioinformatics^18^, pan-cancer analysis has laid the foundation for conducting comprehensive research on tumor molecular characteristics, pathological features, and corresponding clinical attributes across various cancer types 19.

In this study, we performed a thorough analysis utilizing a diverse array of data sources, encompassing TCGA, GTEx, BioGPS, and HPA, to explore the expression patterns, diagnostic implications, and prognostic significance of CCNF across a broad spectrum of malignancies. Additionally, we investigated the interrelation between CCNF expression, DNA methylation profiles, and immune infiltration levels in 33 different cancer types. Moreover, we analyzed the interaction between CCNF and genes as well as targeting chemistries, followed by enrichment analysis of CCNF-related genes. Finally, RT-qpcr and WB assays were employed to assess CCNF expression in established cancer cell lines. Our findings shed light on the potential of CCNF as a promising avenue for novel cancer treatment and prevention strategies.

## Materials and Methods

### Data collection and differential expression analysis

In this research, we obtained RNA sequencing data, somatic mutations, and relevant clinical information, which collectively consisted of 11,321 samples derived from 33 distinct cancer types. These datasets were retrieved from The Cancer Genome Atlas (TCGA) database (https://portal.gdc.cancer.gov/). Additionally, gene expression data pertaining to 31 normal tissues were acquired from the Genotype-Tissue Expression (GTEx) database (https://commonfund.nih.gov/GTEx). And acquired data on CCNF expression in both normal and tumor cell lines from the BioGPS database (http://biogps.org/). Subsequently, normalization and log2 transformation were performed on all datasets. To identify the expression differences between 33 tumor tissues and 31 normal tissues, data analysis was conducted using R software (version 4.0.2, https://www.Rproject.org), and visualization was achieved using the R package "ggplot2."

### Immunohistochemistry Staining (IHC)

For the investigation of CCNF protein-level expression discrepancies in cancer, we obtained immunohistochemistry (IHC) images of 11 tumors and their respective normal tissues from the Human Protein Atlas (HPA) database (http://www.proteinatlas.org/). The tumor types included liver cancer, colorectal cancer, stomach cancer, pancreatic cancer, urothelial cancer, breast cancer, cervical cancer, endometrial cancer, ovarian cancer, melanoma, and lymphoma.

### Analysis of the diagnostic value of CCNF

Utilizing the clinical data obtained from TCGA, we conducted an analysis of CCNF expression concerning clinical phenotype and tumor stage, with subsequent visualization using the "ggplot2" package. To assess the diagnostic efficacy of CCNF, we analyzed and visualized the ROC curves using both the "pROC" package and the "ggplot2" R package.

### Prognostic analysis of CCNF

Based on the survival data obtained from TCGA, which were categorized into overall survival (OS), disease-specific survival (DSS), and progression-free interval (PFI), we employed Kaplan-Meier and univariate Cox regression analyses to investigate the impact of CCNF expression on cancer patient survival. The log-rank test was used to calculate p-values, and hazard ratios (HR) with 95% confidence intervals (95% CI) were derived. The R packages "survival" and "survminer" were utilized for survival analysis and visualization (p < 0.05 for significance). Subsequently, the R package "forestplot" was employed to synthesize the relationship between CCNF expression and pan-cancer survival outcomes.

### Relationship between CCNF expression and immunity

The correlation between CCNF expression and 21 immune infiltrating cell types in pan-cancer was assessed using the TIMER2 database (http://timer.cistrome.org/). This analysis involved the application of XCELL, CIBERSORT-ABS, and EPIC algorithms. Additionally, we conducted a co-expression analysis of CCNF with immune-related genes, encompassing those encoding major histocompatibility complex (MHC) proteins, immune activation markers, immunosuppressive factors, chemokines, and chemokine receptors. The R-package "limma" was employed for this analysis, and the results were visually represented using the "reshape2" and "RColorBrewer" packages.

### Correlation of CCNF expression with DNA methylation

To examine the genetic alterations and mutation rates of CCNF across various cancer types, we utilized the "OncoPrint" and "Cancer Types" modules available in cBioPortal database (https://www.cbioportal.org/). Moreover, we employed the "Summary" modules within cBioPortal to analyze CCNF mutations, expression patterns, and copy number alterations across all samples. Furthermore, the analysis of CCNF promoter methylation in pan-cancer was carried out using UALCAN database (http://ualcan.path.uab.edu/).

### Enrichment analysis of CCNF-related genes

The top 100 genes associated with CCNF and pan-cancer were obtained from the "Similar Genes" module of GEPIA database (http://gepia.cancer-pku.cn/). Subsequently, 20 genes co-expressed with CCNF were identified through the GeneMANIA database (http://www.genemania.org) to predict their functions and investigate their functional similarity and expression patterns to CCNF. The R-packages "clusterProfiler" and "org.Hs.eg.db" were employed for gene ontology analysis, and the results were visualized using bar and bubble plots generated by "ggplot2". Furthermore, to explore differences in gene expression, both activation and repression, among pathway activity groups across 33 cancers, we utilized the "Pathway Activity" module of the GSCALite platform (http://bioinfo.life.hust.edu.cn/web/GSCALite/).

### CCNF interactions with chemicals and genes

The CTD database (http://ctdbase.org/) was used to search for chemicals that interact with CCNF and explore genes highly similar to CCNF in terms of commonly interacting chemicals.

### Cell lines and cell culture

MCF10A, MDA-MB-231, MDA-MB-453, MCF-7, and ZR-75-30, Cells were purchased from ATCC (Manassas, USA). The cells were cultured in DMEM medium supplemented with 10% FBS and incubated under suitable conditions (37°C; 5% CO2).

### Quantitative Real-time PCR (RT-qPCR)

Total cellular RNA was isolated using TRIzol (Vazyme Biotech Co., Ltd), followed by reverse transcription using the HiScript II Q RT SuperMix (Vazyme Biotech Co., Ltd). RT-qPCR detection was carried out using the SYBR Green qPCR kit (Toyobo) on the Real-time PCR Detection instrument (Bio-Rad, USA). The primer sequences employed were as follows: CCNF - Forward primer, 5′-TGTACACTCCCAGCTGAAGGA-3′ and reverse primer, 5′-CCCCTTTTCAGCAGCCCTTT-3′; GAPDH - Forward primer, 5′-GGCCTCCAAGGAGTAAGACC-3′ and reverse primer, 5′-AGGGGTCTACATGGCAACTG-3′. Data analysis was conducted using the 2-ΔΔCT method, and all assays were performed in triplicate.

### Western Blot Assay (WB)

RIPA extraction reagent (Beyotime, China) was used to lyse the cells, and it was treated with protease inhibitors (Sigma-Aldrich, USA). Electrophoresis on polyacrylamide gels containing 10.5%-12.5% sodium dodecyl sulfate separated the total protein, which was then transferred to a 0.45-m PVDF membrane (Millipore, USA). CCNF (P41002, CUSABIO, China) and β-actin (AC006, ABclonal, China) antibodies were primary antibodies. The bands were visualized through the utilization of an enhanced chemiluminescence (ECL) kit (Boster, China) and detected using the ChemiDoc XRS + Imaging System (Bio-Rad, USA).

## Results

### Significant high expression of CCNF in pan-cancer

**Figure 1:**
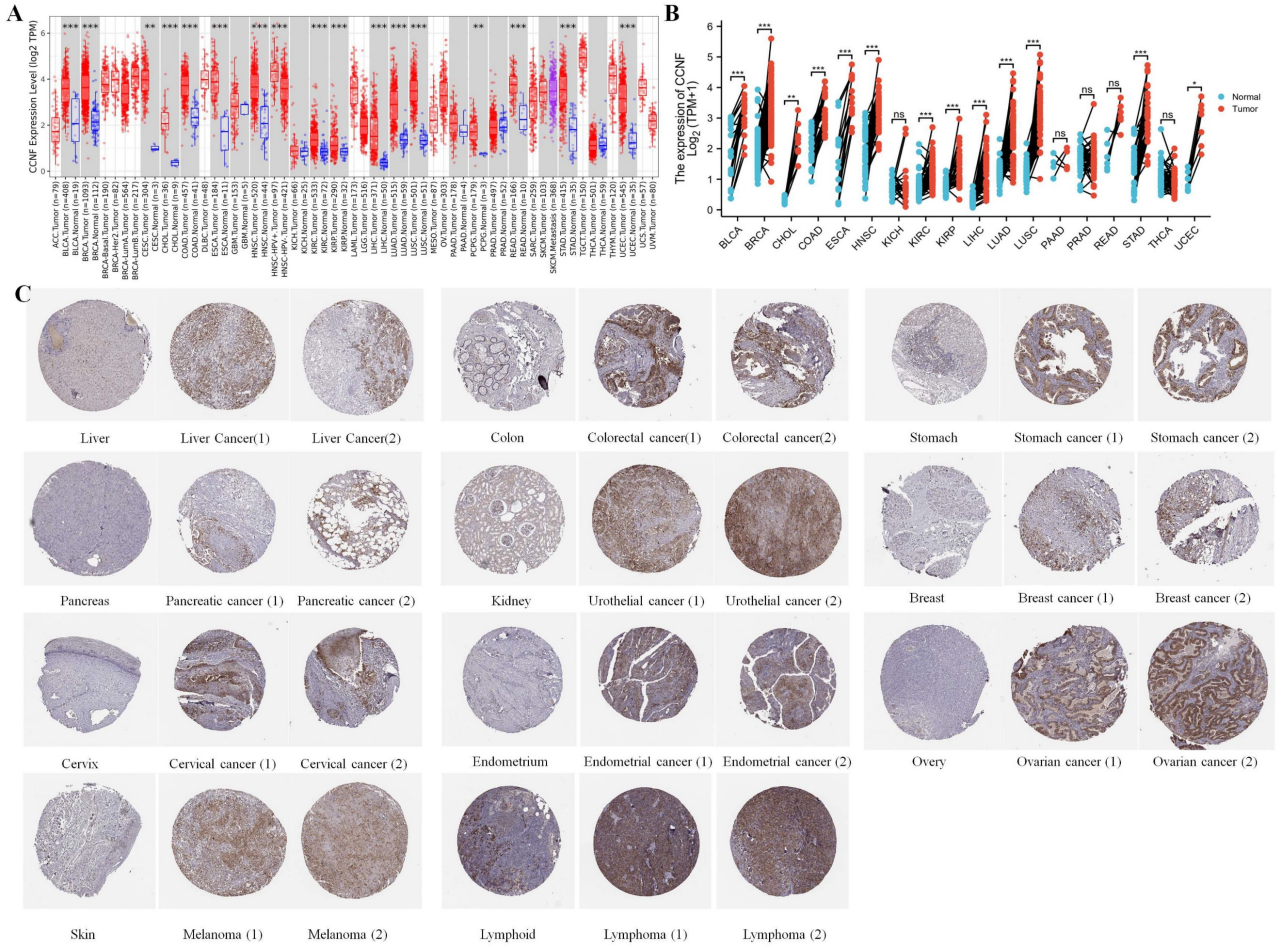
** Expression of CCNF in cancer.** (A) Comparison of CCNF expression between tumor and normal samples. (B) Comparison of CCNF expression between tumor and paired normal samples. (C) The protein expression of CCNF in immunohistochemical images of normal (left) and tumor (right) groups. (ns, p ≥ 0.05; ∗, p < 0.05; ∗∗, p < 0.01; ∗∗∗, p < 0.001).

Based on the analysis of normal tissues in the GTEx database, we found that CCNF had the highest expression level in esophageal tissues and the lowest expression level in heart muscle tissues (Figure S1A). In contrast, upon examining cancer tissues from the TCGA database, CCNF exhibited the highest expression in Testicular Germ Cell Tumors (TGCT), while Kidney Chromophobe (KICH) displayed both the highest and lowest expression levels (Figure S1B). Furthermore, by assessing the expression of CCNF in normal and cancer cell lines from the BioGPS database, we observed that the overall expression level of CCNF was generally higher in cancer cell lines compared to normal cell lines (Figure S1C, S1D). Compared to the normal tissues of GTEx, CCNF expression exhibited significant upregulation in 16 cancer types (Figure 1A), encompassing bladder urothelial carcinoma (BLCA), breast invasive carcinoma (BRCA), cholangiocarcinoma (CHOL), colon adenocarcinoma (COAD), esophageal carcinoma (ESCA), head and neck squamous cell carcinoma (HNSC), kidney renal clear cell carcinoma (KIRC), kidney renal papillary cell carcinoma (KIRP), liver hepatocellular carcinoma (LIHC), lung adenocarcinoma (LUAD), lung squamous cell carcinoma (LUSC), cervical squamous cell carcinoma and endocervical adenocarcinoma (CESC), rectum adenocarcinoma (READ), stomach adenocarcinoma (STAD), and uterine Corpus Endometrial Carcinoma (UCEC) (all p < 0.001), Pheochromocytoma and Paraganglioma (PCPG) (p < 0.01). Moreover, in HPV-positive head and neck squamous cell tumor tissues, CCNF expression was markedly higher compared to HPV-negative tissues (p < 0.001). Differential expression analysis of paired samples demonstrated significant upregulation of CCNF expression in BLCA, BRCA, CHOL, COAD, ESCA, HNSC, KIRC, KIRP, LIHC, LUAD, LUSC, STAD, and UCEC (Figure 1B).

Furthermore, we examined the expression of CCNF protein levels by analyzing the immunohistochemistry (IHC) results of both normal and tumor tissues. The findings revealed elevated CCNF expression in 11 tumor tissues compared to normal tissues (Figure 1C).

### Diagnostic value of CCNF in pan-cancer

**Figure 2:**
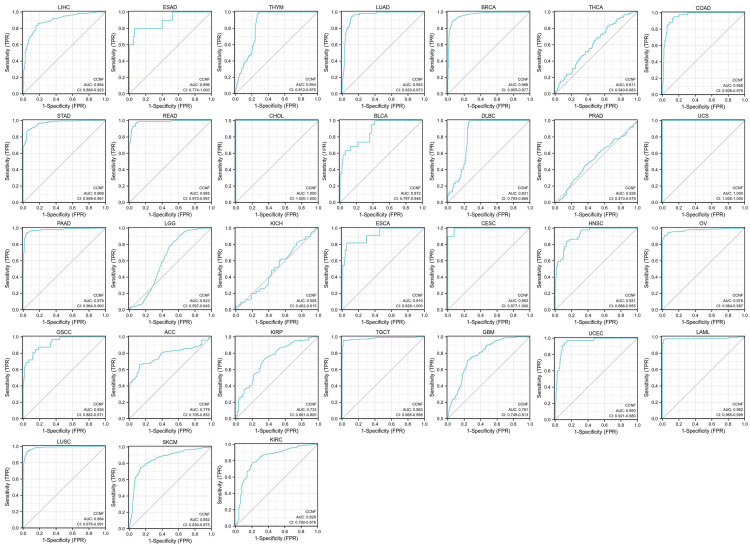
** AUC of ROC curves verified the diagnosis performance of CCNF.** AUC of ROC curves verified the diagnosis performance of CCNF in the TCGA cohort, high diagnostic accuracy (AUC: 1.0-0.9), relative diagnostic accuracy (AUC: 0.9-0.7), and Low diagnostic accuracy (AUC: 0.7-0.5).

We conducted an investigation into the clinical correlation of CCNF expression based on TNM staging (T: tumor size, N: lymph node involvement, M: distant metastasis). The analysis revealed a significant increase in CCNF expression at early stages in 15 cancers (Figure S2), namely, BLCA, BRCA, CHOL, COAD, ESCA, HNSC, KIRC, LIHC, LUAD, LUSC, STAD, KIRP, READ, thyroid carcinoma (THCA), and oral squamous cell carcinoma (OSCC). These findings suggest the potential of CCNF as a valuable marker for early tumor diagnosis in a wide range of cancers (Figure S2). Additionally, the receiver operating characteristic (ROC) analysis demonstrated high diagnostic accuracy for CCNF in 17 cancers, relative diagnostic accuracy in 10 cancers, and low diagnostic accuracy in 4 cancers. Notably, the area under the curve (AUC) reached 1.0 in CHOL and UCS (Figure 2).

### Prognostic role of CCNF in pan-cancer

**Figure 3:**
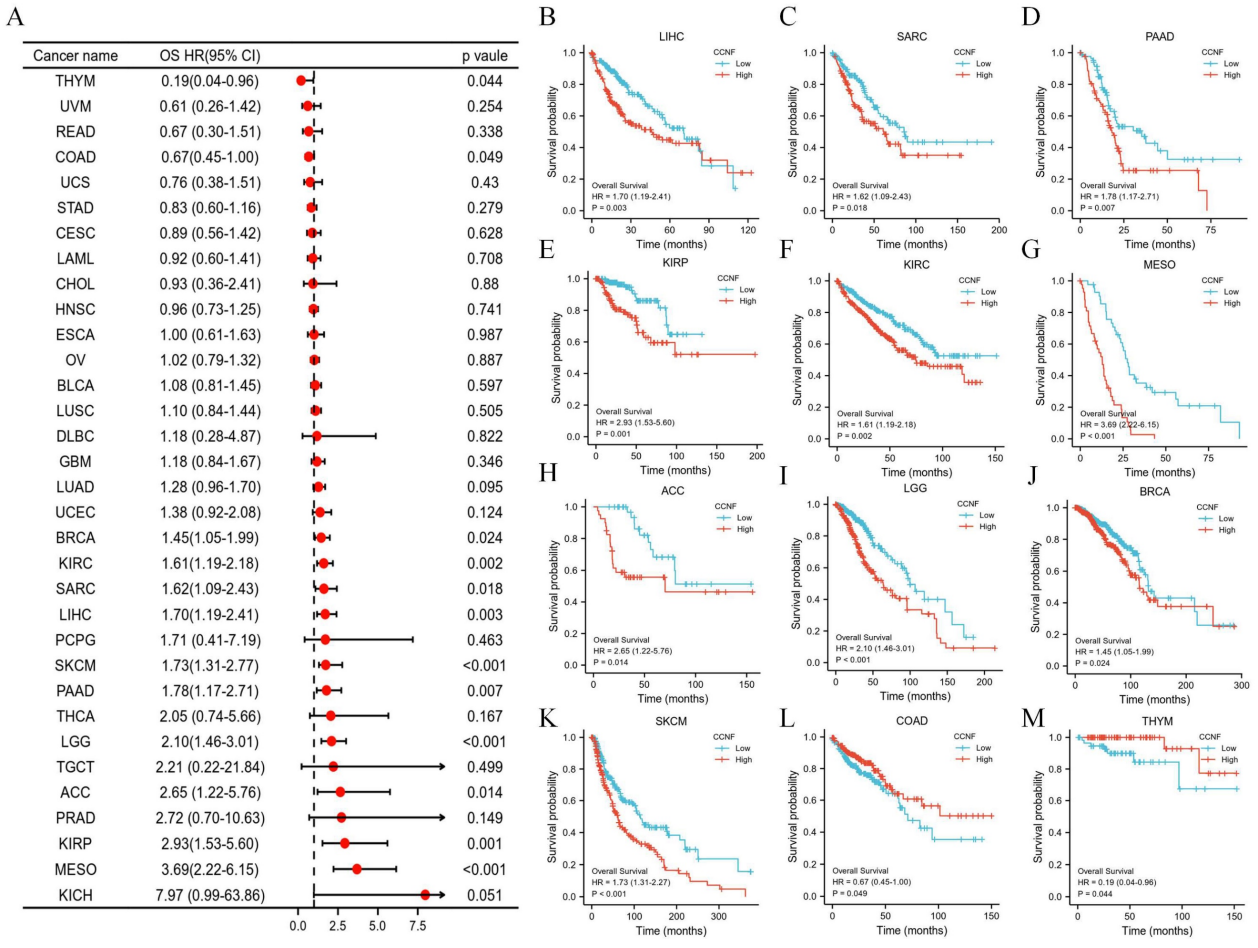
** Association between CCNF expression and overall survival (OS).** (A) Forest plot of OS associations in different tumor types. (B-N) Kaplan-Meier analysis of the association between CCNF expression and OS.

To investigate the prognostic significance of CCNF across different cancer types, we conducted survival association analyses, as well as univariate Cox regression analyses for each cancer. The results indicated that CCNF played a significant role in the prognosis of several cancers, such as breast invasive carcinoma (BRCA), kidney renal clear cell carcinoma (KIRC), liver hepatocellular carcinoma (LIHC), sarcoma (SARC), kidney renal papillary cell carcinoma (KIRP), pancreatic adenocarcinoma (PAAD), mesothelioma (MESO), brain lower grade glioma (LGG), skin cutaneous melanoma (SKCM), and adrenocortical carcinoma (ACC), acting as a high-risk factor. Conversely, CCNF served as a low-risk factor in colon adenocarcinoma (COAD) and thymoma (THYM) (Figure 3A). Kaplan-Meier survival analysis further demonstrated that high CCNF expression was associated with poor overall survival (OS) in patients with BRCA, KIRC, LIHC, SARC, KIRP, PAAD, MESO, LGG, SKCM, and ACC (Figure 3B-K), while high CCNF expression correlated with favorable patient outcomes in COAD and THYM (Figure 3L-M). Moreover, for disease-specific survival (DSS), high CCNF expression significantly contributed to poorer clinical outcomes in KIRC, LIHC, KIRP, LGG, ACC, lung adenocarcinoma (LUAD), MESO, and SKCM (Figure S3). Similarly, for progression-free interval (PFI), CCNF was significantly associated with worse clinical outcomes in KIRC, LIHC, KIRP, LGG, ACC, PAAD, prostate adenocarcinoma (PRAD), and MESO, while acting as a protective factor in COAD (Figure S4).

### Relationship between CCNF and immune infiltration

**Figure 4:**
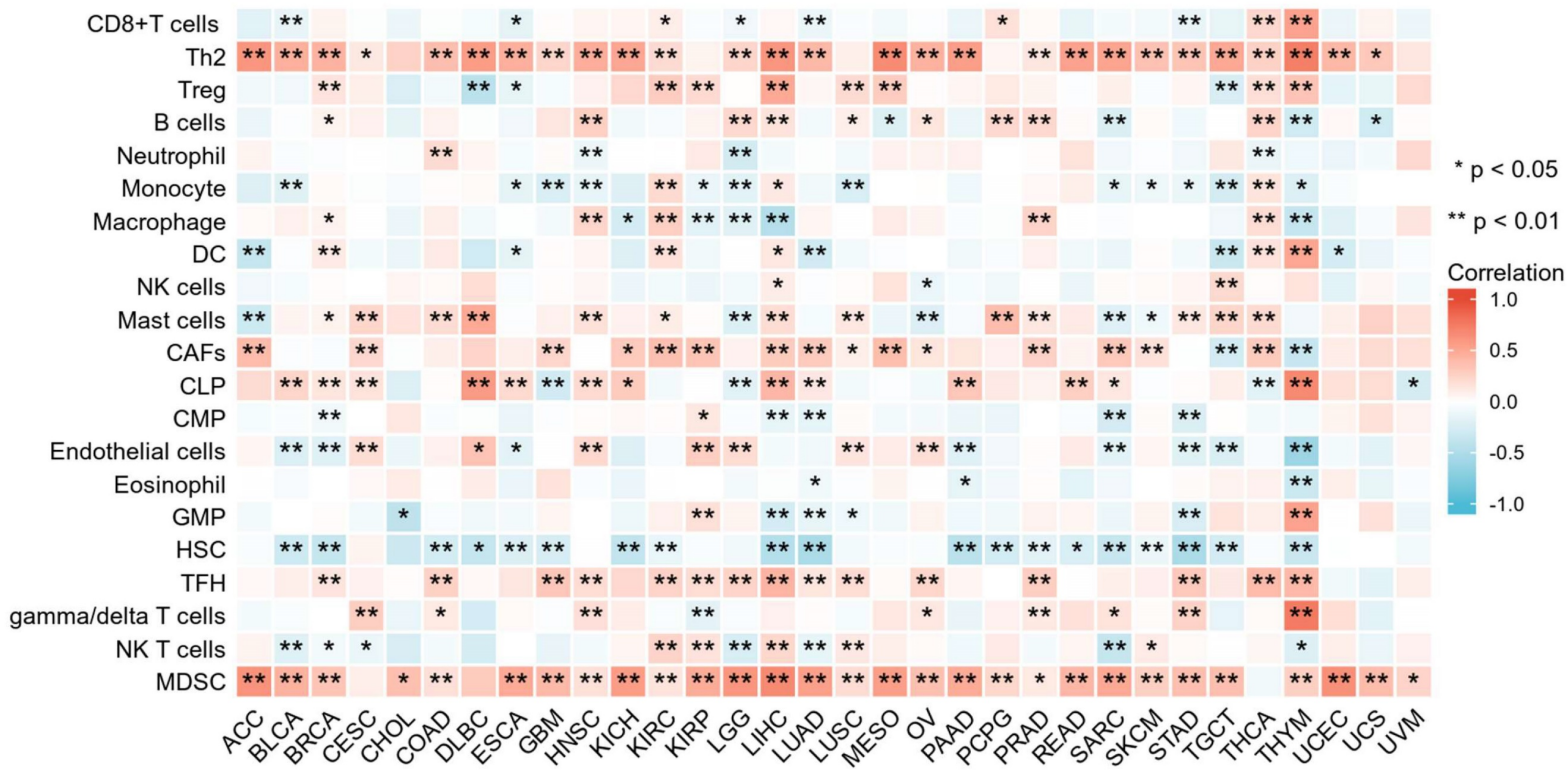
** Relationship between CCNF expression and immune cell infiltration in different cancers.** Correlation between CCNF expression and different immune cells from TIMER2 database. Red represents positive correlation, blue represents negative correlation, and the darker the color, the stronger the correlation. *p < 0.05, **p < 0.01.

The correlation between CCNF expression and tumor immune cell infiltration was evaluated using the TIMER2 database. The results demonstrated that CCNF expression exhibited negative correlations with immune cells in most tumors, while also showing significant positive correlations in certain cancers. For instance, in thymoma (THYM), CCNF expression levels exhibited significant positive correlations with Th2 cells, common lymphoid progenitor (CLP), and gamma/delta T cells. Moreover, CCNF expression levels positively correlated with Th2 cells, cancer-associated fibroblast (CAFs), follicular helper T cells (Tfh), and myeloid-derived suppressor cells (MDSC) across various immune cells (Figure 4). Figure S5 demonstrates the four cancers with the strongest association between CCNF and Th2 cells (Figure S5A), follicular helper T cells (Figure S5B) and MDSC cells (Figure S5C).

Furthermore, we explored the association between CCNF expression and immune-related genes in pan-cancer. The co-expression analysis revealed that, in most cancers, CCNF expression positively correlated with genes related to chemokines (Figure S6A), immune activation (Figure S6B), chemokine receptors (Figure S6C), major histocompatibility complex (MHC) (Figure S6D), and immunosuppression (Figure S6E). These findings suggest that CCNF expression may impact tumor immune infiltration through its influence on these immune-related factors.

### The impact of CCNF on DNA methylation in cancer

The expression of DNA methylation-regulated genes is closely associated with cancer, and we calculated the promoter methylation levels of CCNF in pan-cancer using the UALCAN database. As depicted in Figure S7, the promoter methylation levels of CCNF were significantly lower in ESCA, CESC, SARC, PAAD, BLCA, BRCA, LUSC, HNSC, READ, PCPG, STAD, TGCT, CHOL, and KIRC compared to the normal group. While the promoter methylation level of CCNF in LUAD was notably lower than in the normal group. Furthermore, data from the CTD database revealed chemicals affecting CCNF methylation (Table 1), including Aflatoxin B1, aflatoxin B2, benzo(e)pyrene, Methapyrilene, and Valproic Acid, which increased the methylation level of CCNF in cancer. Conversely, Arsenic Trioxide was found to down-regulate the methylation level of CCNF.

We conducted further exploration of the genetic alterations of CCNF in pan-cancer utilizing the cBioPortal database. The most prevalent mutation types observed were Missense mutations, Amplifications, and Deep Deletions. Notably, CCNF mutations were particularly frequent in adrenocortical carcinoma (ACC), mesothelioma (MESO), breast invasive carcinoma (BRCA), esophageal carcinoma (ESCA), uterine corpus endometrial carcinoma (UCEC), bladder urothelial carcinoma (BLCA), lymphoid neoplasm diffuse large B-cell lymphoma (DLBC), and cervical squamous cell carcinoma and endocervical adenocarcinoma (CESC) (Figure S8A). Additionally, the mutation types of CCNF were further analyzed using the "OncoPrint" module, revealing that CCNF exhibited genetic alterations in 2% of cancer cases, primarily consisting of Amplifications and Deep Deletions, while other alterations were not as significant (Figure S8B).

### Enrichment analysis of CCNF-related genes

To explore the biological significance of CCNF expression in pan-cancer, we retrieved 100 genes with similarity to CCNF from the GEPIA2 database (Table S1). Additionally, 20 co-expressed genes with CCNF were obtained from the GeneMANIA database (Figure S9A), among which CCP110 exhibited the most significant correlation with CCNF. Functional analysis revealed that CCNF and its similar genes were primarily associated with chromosome segregation, cell cycle regulation, and nuclear division. We conducted GO and KEGG pathway analyses using genes from the GEPIA2 and GeneMANIA databases. The GO enrichment analysis demonstrated that biological processes (BP) were predominantly enriched in chromosome segregation, mitosis, and nuclear division. The cellular component (CC) was enriched in the chromosomal region, centromeric region, and spindle. The molecular function (MF) was enriched in microtubule binding, motor activity, ATPase activity, among others. In the KEGG pathways, the main associations were observed with the Cell cycle, Cellular senescence, p53 signaling pathway, DNA replication, FoxO signaling pathway, and MicroRNAs in cancer (Figure S9C).

Next, we employed the GSCALite database to investigate the impact of gene sets on tumor pathway activity. The analysis included the first four genes associated with CCNF (PLK1, KIF2C, DLGAP5, and NCAPH). The pie chart demonstrated that CCNF had activating effects on DNA damage response, Cell cycle, Apoptosis, and TSC/mTOR pathways, while exhibiting inhibitory effects on Ras/MAPK and Hormone ER pathways (Figure S9D). Additionally, we further analyzed the percentage of potential genomic effects on pathway activity. The results showed that CCNF activated 69% of the cell cycle and 25% of apoptosis in cancer, while inhibiting 18% of the Ras/MAPK and Hormone ER pathways in cancer (Figure S9E). These findings collectively suggest that CCNF may influence tumorigenic progression by modulating these biological processes.

### Chemicals and genes interacting with CCNF

A total of 143 chemicals associated with CCNF were retrieved from the CTD database, with 48 chemicals exhibiting upregulation effects on CCNF, while 77 chemicals demonstrated downregulation effects. Among these, 18 chemicals were identified to influence CCNF expression, but their specific effects require further validation (Table S2).

Subsequently, we investigated 20 genes that displayed similarity to CCNF based on chemical association. The findings revealed that CCNF shares similar drug targets and sensitivity with these genes (Table S3).

### CCNF expression in breast cancer cell lines

**Figure 5:**
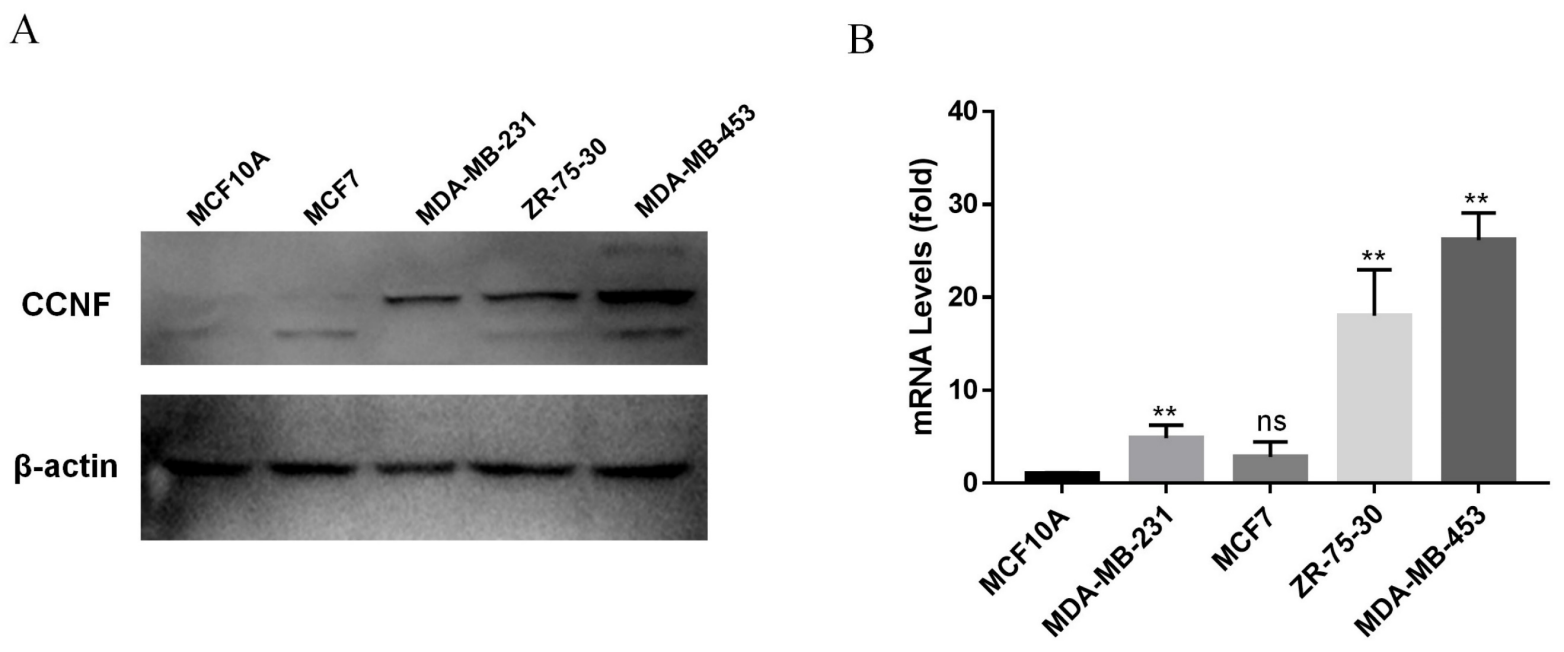
** Expression of CCNF in breast cancer cell lines.** (A) Real-time real-time quantitative polymerase chain reaction (RT-qPCR) was used to analyze the expression of CCNF (ns, p ≥ 0.05; ∗∗, p < 0.01). (B) Western blotting (WB) was used to detect the relative expression of CCNF (compared to GAPDH).

To investigate the elevated expression of CCNF in tumors, we focused on breast cancer, which serves as a representative human cancer type, for further investigation. Through qRT-PCR and WB analyses, it was observed that CCNF exhibited significant upregulation in breast cancer cells, namely MCF-7, MDA-MB-453, ZR-75-30, and SK-BR-3, when compared to the normal breast cancer cell line MCF10A (Figure 5A, B).

## Discussion

CCNF is an important factor involved in regulating apoptosis, angiogenesis, and epithelial-mesenchymal transition (EMT). Increasing evidence suggests that its dysregulation can contribute to the evasion of apoptosis, promotion of angiogenesis, and acquisition of invasiveness by cancer cells 20, 21. In this study, we conducted a comprehensive multi-omics analysis to investigate the intricate association between CCNF and pan-cancer. Our investigation encompassed various aspects, including mRNA expression, diagnostic potential, DNA methylation, mutation profiles, immune infiltration levels, and prognostic implications for patients. Furthermore, we employed functional enrichment analysis to elucidate the underlying biological functions and pathways implicated in CCNF-mediated tumorigenesis. Furthermore, we verified the high expression of CCNF in breast cancer by RT-qPCR and WB analysis.

Our findings revealed significant upregulation of CCNF mRNA expression in 16 cancer types, namely BLCA, BRCA, CHOL, COAD, ESCA, HNSC, KIRC, KIRP, LIHC, LUAD, LUSC, CESC, READ, STAD, UCEC, and PCPG. And among them, the expression of CCNF was significantly increased in paired samples of 13 cancers. Furthermore, IHC images showed that in liver cancer, colorectal cancer, stomach cancer, pancreatic cancer, urothelial cancer, breast cancer, cervical cancer, endometrial cancer, ovarian cancer, melanoma and lymphoma tissues, the protein expression level of CCNF was higher than that of normal tissues. It is noteworthy that the protein expression levels in liver cancer, gastric cancer, pancreatic cancer, ovarian cancer, melanoma, and lymphoma did not consistently correlate with mRNA expression. This discrepancy may arise from the complexity of gene regulation, where mRNA molecules can produce multiple polypeptides and transcription rates can vary in space and time 22. Moreover, proteins undergo regulation at multiple levels, encompassing transcription, mRNA processing, and translation, which can result in variations between mRNA and protein expression levels 23. Previous studies have demonstrated elevated CCNF expression in breast cancer 16, melanoma 24, colorectal cancer 25, gastric cancer 26, ovarian cancer 15, liver cancer 27, and pancreatic cancer 28, and g which were associated with unfavorable patient outcomes. These findings are in line with our results, reinforcing the validity and reliability of our study. In contrast, Chen et al. reported an association between CCNF expression levels and improved prognostic outcomes in colorectal cancer 29, Fu et al. observed that high CCNF mRNA and protein expression were linked to better prognosis in HCC patients 17. Additionally, Chang et al. identified downregulated CCNF protein expression in breast cancer tissues, with CCNF overexpression inhibiting breast cancer cell growth 30. These variations could be influenced by tumor subtypes, differences in samples, and biological heterogeneity. Hence, further investigations are necessary to elucidate the specific roles and mechanisms of CCNF in relation to tumors.

Early detection and timely treatment are crucial to the prognosis of cancer patients, emphasizing the significance of early cancer diagnosis. In this study, we investigated the differential expression of CCNF across various tumor stages to assess its potential as a diagnostic marker. Notably, our ROC analysis demonstrated high diagnostic accuracy of CCNF in 17 cancers, with particularly notable performance in CHOL and UCS. Additionally, we examined the association between CCNF expression and tumor prognosis using Cox regression and Kaplan-Meier survival analysis. The results indicated that high CCNF expression was linked to favorable prognosis in KIRC, LIHC, KIRP, PAAD, MESO, LGG, and ACC, while it indicated poor prognosis in COAD. Furthermore, our analysis demonstrated a significant increase in CCNF expression during the early stages of BLCA, BRCA, CHOL, COAD, ESCA, HNSC, KIRC, LIHC, LUAD, LUSC, STAD, KIRP, READ, THCA, and OSCC.

The tumor immune microenvironment (TIME) plays a crucial role in cancer development 31, and improving TIME contributes to cancer immunotherapy 32. The TME primarily consists of cancer-associated fibroblasts (CAFs), tumor cells, tumor-infiltrating immune cells, endothelial cells, extracellular matrix (ECM), and various signaling molecules 33. To comprehend the role of CCNF in the tumor microenvironment, we investigated its correlation with tumor-infiltrating immune cells. Our findings revealed significant associations between CCNF expression and infiltration levels of multiple tumor immune cells, particularly Th2 cells, CAFs, Tfh cells, and MDSCs across various tumor types. CAFs, a heterogeneous population derived from various cell origins 34, exert immunosuppressive effects in the TIME by secreting diverse cytokines, growth factors, chemokines, and exosomes. These factors interact with tumor-infiltrating immune cells and other immune components within the TIME, enabling cancer cells to evade immune system recognition 35, 36. Th2 and Tfh cells belong to the T cell subpopulation and differentiate from naive CD4+ T cells 37, CAFs promote the conversion of naive T cells into an immunosuppressive subpopulation in tumors, suppressing the activity of effector T lymphocytes and producing immunosuppression in the TME 38. Furthermore, MDSC, an immunosuppressive cell that is recruited in large numbers in tumors 39, play a role in promoting cancer invasion, metastasis, and immune tolerance 40, 41. Additionally, CAF-modified MDSCs are significantly increased in the TIME, further enhancing tumor immunosuppression 42. Subsequently, we investigated the impact of CCNF expression on immune-related genes, including major histocompatibility complex (MHC) genes, immunosuppressive genes, chemokines, and chemokine receptors. Our findings revealed significant co-expression between CCNF and the majority of immune-related genes. Particularly, CCNF expression exhibited a strong positive correlation with genes like CCR10, TAP1, TAP2, PVR, MICB, TGFBR1, IL10RB, and ADORA2A. These observations suggest that CCNF may interact with immune infiltrating cells, triggering the secretion of these cellular components by CAFs, thus fostering tumor immune evasion. In conclusion, CCNF expression level significantly influences the TIME and provides valuable insights for the development of tumor immunotherapy strategies.

DNA methylation, an essential epigenetic modification, plays a pivotal role in regulating gene expression without altering the DNA sequence 43. Aberrant promoter methylation is widely recognized as a hallmark of cancer and involves the silencing of tumor suppressor genes and the activation of oncogenes 44. In this study, we observed that high CCNF expression in most cancer types is associated with reduced DNA methylation, which can result in tumor genomic hypomethylation. This hypomethylation is known to reduce chromosomal stability and contribute to abnormal tumor metabolism and differentiation, which are directly related to the development of tumors 45, 46. In addition, chemicals impacting CCNF methylation were obtained through the CTD database, and these findings may be helpful for methylation therapy in cancer. However, the specific mechanisms underlying DNA hypomethylation in cancer are currently a subject of extensive debate, necessitating further investigation to elucidate the intricate relationship between DNA methylation and CCNF expression.

Enrichment analysis revealed that CCNF may exert its influence on cancer pathogenesis through various pathways, including cell cycle-related pathways, cellular senescence-related pathways, the FoxO signaling pathway, and the P53 signaling pathway. Consistent with previous studies, CCNF has been shown to regulate normal cell cycle progression and maintain genomic stability 13. Notably, high CCNF expression has been implicated in both promoting and suppressing cancer, underscoring its significant role in cancer growth and metabolism 47. By leveraging the GeneMANIA and GEPIA2 databases, we identified co-expressed genes associated with CCNF. Additionally, we observed a significant correlation between CCNF expression and two key signaling pathways: the FoxO signaling pathway and the P53 cell signaling pathway. FOXO proteins, acting as transcription factors, are tightly regulated by growth factors and stress 48, and they play multifaceted roles in cancer development and metastasis, including processes such as cell differentiation, apoptosis, cell proliferation, DNA damage, and repair 49 50. On the other hand, P53, encoded by the TP53 gene 51, is a vital tumor suppressor that plays diverse roles in cellular stress response, such as regulating cell cycle, DNA damage response, and apoptosis 52. The presence of TP53 mutations is frequently observed across various cancers, leading to reduced antitumor activity and, paradoxically, conferring oncogenic effects to the p53 protein 53. Moreover, our investigation delved into the pathways associated with gene expression activation and inhibition in cancer. Notably, CCNF expression was found to activate pathways related to DNA damage response, cell cycle regulation, apoptosis, and the TSC/mTOR pathway, while inhibiting the Ras/MAPK and Hormone ER pathways. This suggests that CCNF-related genes may exert their influence on cancer through multiple mechanisms. It is important to note that the up- or down-regulation of these signaling pathways by CCNF may exhibit variation across different cancer types, warranting further exploration into the specific mechanisms underlying their regulation of cancer.

Moreover, we validated the upregulated expression of CCNF in breast cancer cells through qRT-PCR and WB, corroborating the findings reported by Liu et al. 16. In conclusion, our study provides further evidence supporting the oncogenic role of elevated CCNF expression in cancer and emphasizes its potential as a promising target for tumor therapy.

## Summary and Prospects

In this study, we investigated the potential role and mechanism of CCNF in pan-cancer using using an advanced pan-cancer analysis system. The results unveiled distinct expression patterns of CCNF between tumor and normal tissues. Furthermore, we experimentally verified the high expression of CCNF in breast cancer cells, establishing its correlation with clinical diagnosis, prognosis, and DNA methylation. Moreover, through immune infiltration and enrichment analyses, we uncovered the contribution of elevated CCNF expression to the tumor immunosuppressive microenvironment. Additionally, our findings indicated that CCNF may exert its regulatory effect on cancer development through the cell cycle, FoxO signaling, and P53 signaling pathways. However, it is important to acknowledge the limitations of this study. Firstly, the small sample size of certain tumors in the database and discrepancies in sequencing methods may compromise the accuracy of the data. Secondly, our study solely provides preliminary evidence of the association between CCNF and pan-cancer. Further experiments are warranted to elucidate the specific molecular functions and mechanisms of CCNF in tumorigenesis. Nonetheless, our study lays a significant groundwork for comprehending the role of CCNF in cancer and provides valuable insights for the future development of precise targeted therapies and immunotherapy.

## Supplementary Material

Supplementary figures and tables.

## Figures and Tables

**Table 1:** Chemicals that affect CCNF methylation

Chemical Name	Chemical ID	Interaction Actions
Aflatoxin B1	D016604	Increases methylation
aflatoxin B2	C029753	Increases methylation
Arsenic Trioxide	D000077237	Decreases methylation
benzo(e)pyrene	C026487	Increases methylation
Methapyrilene	D008701	Increases methylation
Valproic Acid	D014635	Increases methylation

## Abbreviations

ACC: adrenocortical carcinoma
BLCA: bladder Urothelial Carcinoma
BRCA: breast invasive carcinoma
CESC: cervical squamous cell carcinoma and endocervical adenocarcinoma
CHOL: cholangiocarcinoma
COAD: colon adenocarcinoma
DLBC: lymphoid neoplasm diffuse large B-cell lymphoma
ESCA: esophageal carcinoma
GBM: glioblastoma multiforme
HNSC: head and Neck squamous cell carcinoma
KICH: kidney Chromophobe
KIRC: kidney renal clear cell carcinoma
KIRP: kidney renal papillary cell carcinoma
LAML: acute Myeloid Leukemia
LGG: lower Grade Glioma
HCC/LIHC: liver hepatocellular carcinoma
LUAD: lung adenocarcinoma
LUSC: lung squamous cell carcinoma
MESO: mesothelioma
OV: ovarian serous cystadenocarcinoma
PAAD: pancreatic adenocarcinoma
PCPG: pheochromocytoma and Paraganglioma
PRAD: prostate adenocarcinoma
READ: rectum adenocarcinoma
SARC: sarcoma
SKCM: skin Cutaneous Melanoma
STAD: stomach adenocarcinoma
TGCT: testicular Germ Cell Tumor
THCA: thyroid carcinoma
THYM: thymoma
UCEC: uterine Corpus Endometrial Carcinoma
UCS: uterine Carcinosarcoma
UVM: uveal Melanoma
TCGA: The Cancer Genome Atlas
GTEx: Genotype-Tissue Expression
