# Supplementary material for: A Comprehensive Analysis of the Prognostic, Immunological and Diagnostic Role of CCNF in Pan-cancer
Source: J Cancer. 2023 Jul 31;14(13):2431–42. doi: 10.7150/jca.86597 (PMC10475360; doi:10.7150/jca.86597)
Supplement: Supplementary file 1 — Supplementary figures and tables. [file jcav14p2431s1.pdf]

Title: A Comprehensive Analysis of the Prognostic, Immunological and Diagnostic Role of CCNF in pan-carcinoma

Running Head: A Comprehensive Analysis of CCNF in pan-carcinoma

Gao Xiaofeng[1, 2, *], Bu Huitong[3, *], Gao Xuzheng[1], Wang Ying[1], Zhang zhenwang[1, 2#], Wang Long[1, 2, 4,#]

(1. *School of Basic Medical Sciences, Xianning Medical College*, *Hubei University of Science and Technology, Hubei University of Science and Technology*,

2. *Medicine Research Institute /Hubei provincial key laboratory of diabetic cardiovascular diseases*, *Xianning Medical College, Hubei University of Science and Technology, Hubei Xianning* 437100,

3. College of Biology, Hunan University, Hunan Changsha, 410012,

4. School of Stomatology and Ophthalmology, Xianning Medical College, Hubei University of Science and Technology, Xianning 437100, Hubei, China 437100)

#Corresponding Author:

Ph.D. Wang Long

School of Basic Medical Sciences, Xianning Medical College, Hubei University of Science and Technology, Xianning 437100, Hubei, China. Email: wanglong@hbust.edu.cn

Ph.D. Zhang zhenwang

Medicine Research Institute / Hubei Key Laboratory of Diabetes and Angiopathy, Xianning Medical College, Hubei University of Science and Technology, Xianning 437100, Hubei, China. Email: zhenwangzhang@hbust.edu.cn, Tel +86 0715-8236051

Supplementary Figure 1.

Figure S1

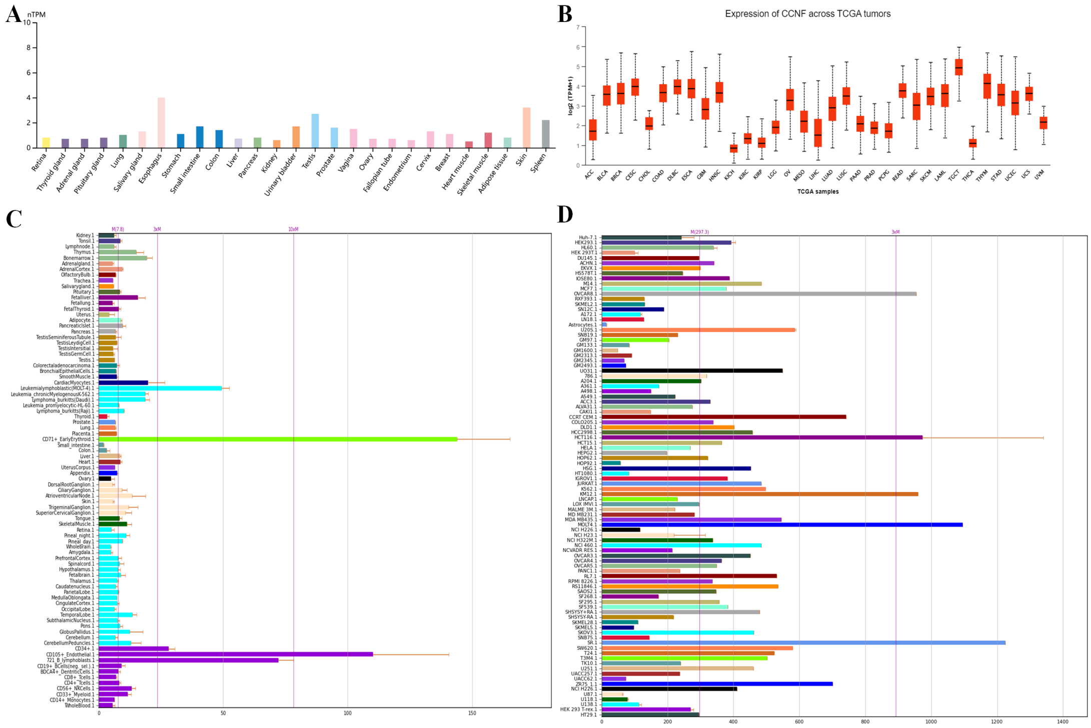

Figure S1. Differential expression of CCNF in cell lines and tissues.

(A) Expression of CCNF in normal tissues. (B) Expression of CCNF in 33 types of cancer. (C) Expression of CCNF in cancer cell lines. (D) Expression of CCNF in normal cell lines.

Figure S2

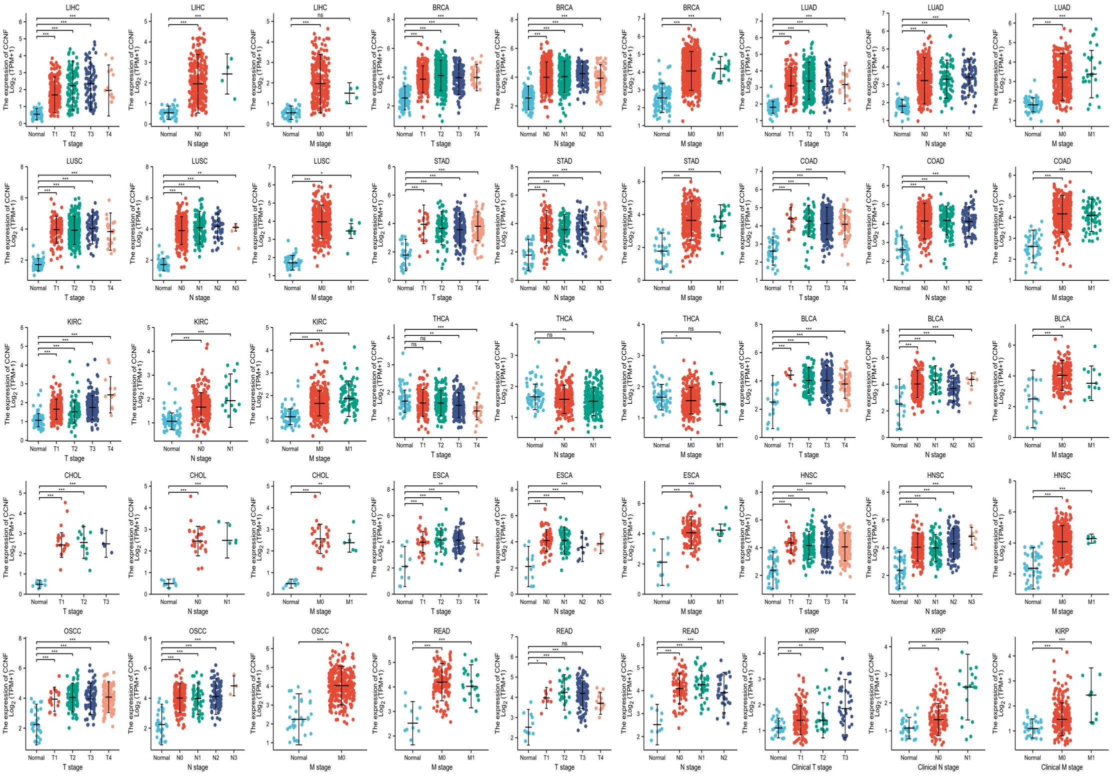

Figure S2. Relationship between CCNF expression and clinicopathological features.

The expression levels of CCNF in the clinical stages of 15 tumors and corresponding normal tissues. *p < 0.05, ***p* < 0.01, ****p* < 0.001. ns, not statistically significant.

Figure S3

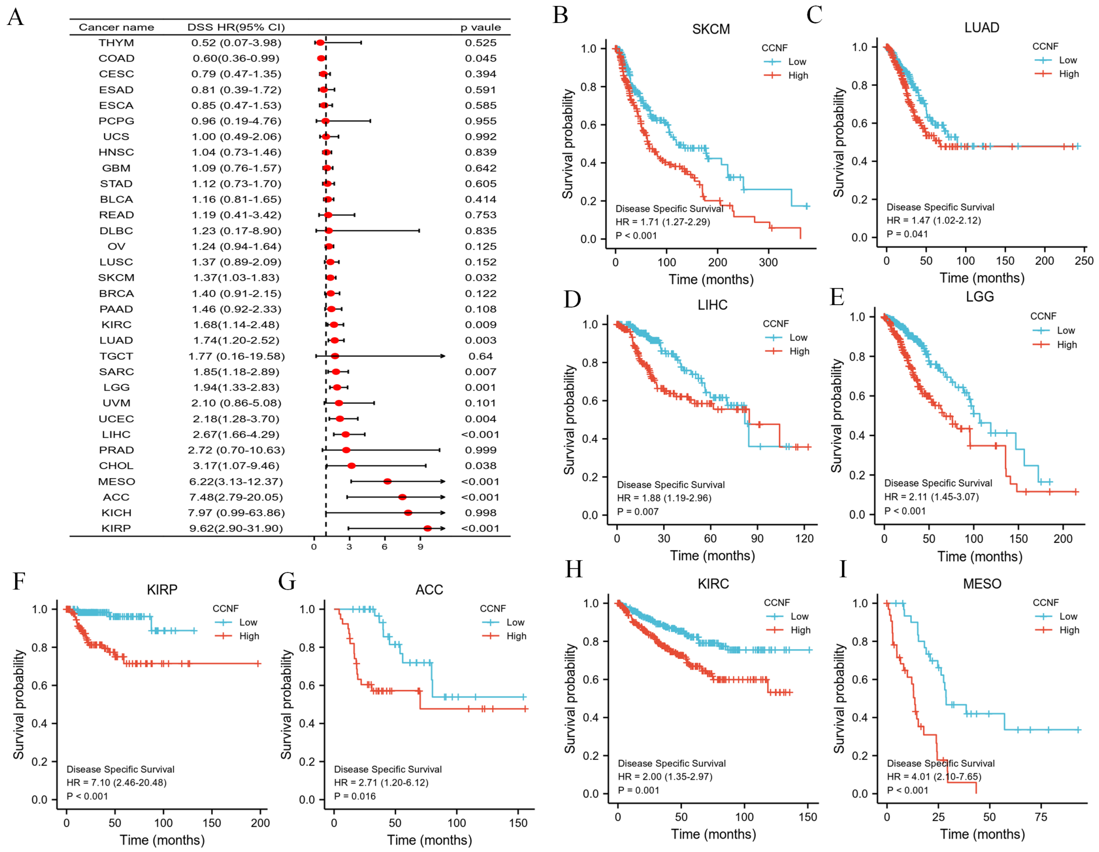

Figure S3. Association between CCNF expression and disease-specific survival (DSS).
(A) Forest plot of DSS associations in different tumor types. (B–K) Kaplan–Meier analysis of the association between CCNF expression and DSS.

Figure S4

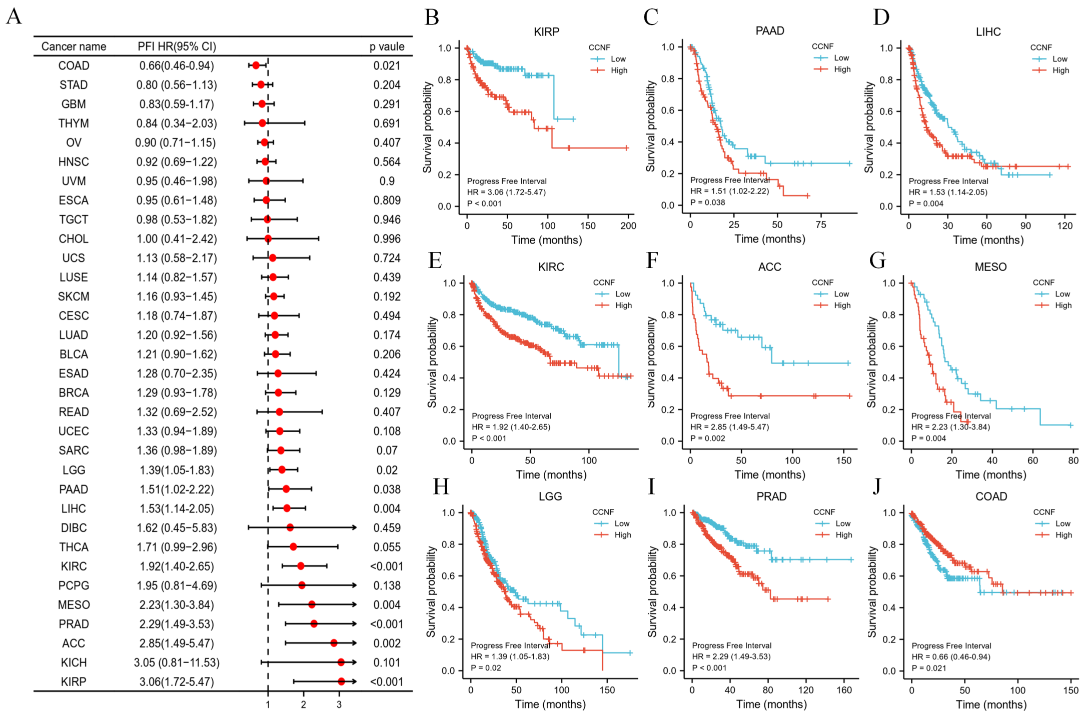

Figure S4. Association between CCNF expression levels and progression-free interval (PFI). (A) Forest plot of PFI associations in different tumor types. (B–S) Kaplan–Meier analysis of the association between CCNF expression and PFI.

Figure S5

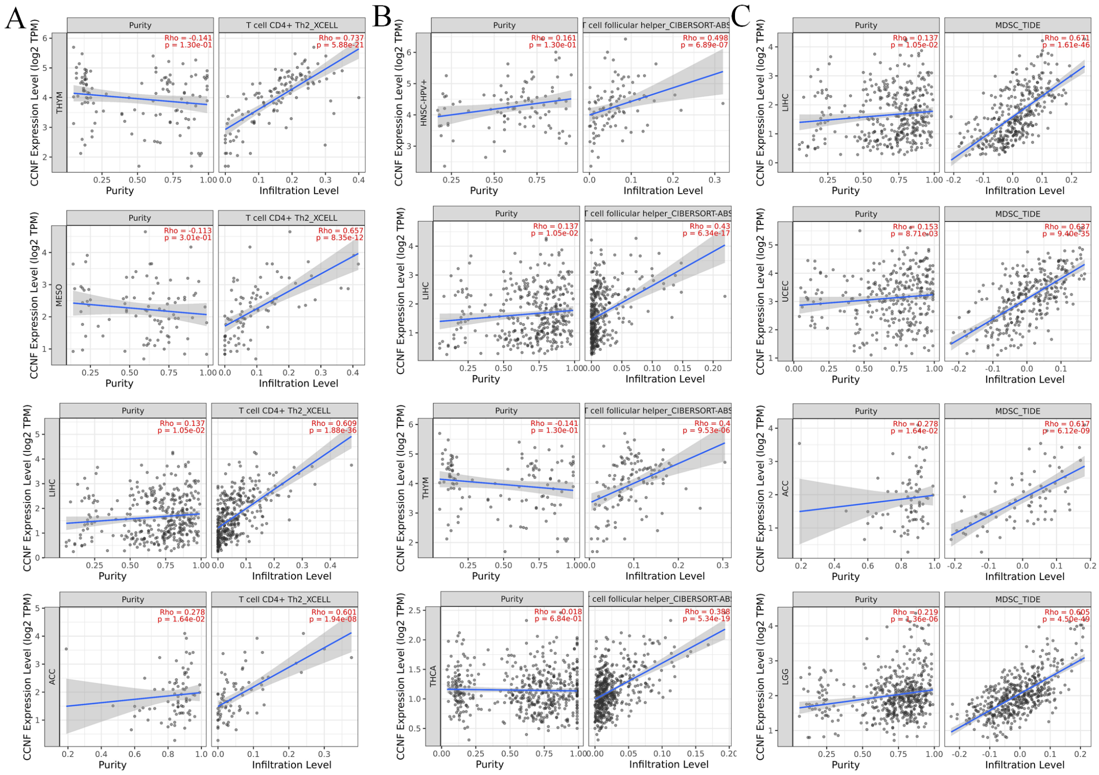

Figure S5. The four tumors with the highest correlation coefficient between CCNF expression and tumor immune infiltrating cells.

(A) Th2 cells. (B) Follicular helper T cell. (C) MDSC.

Figure S6

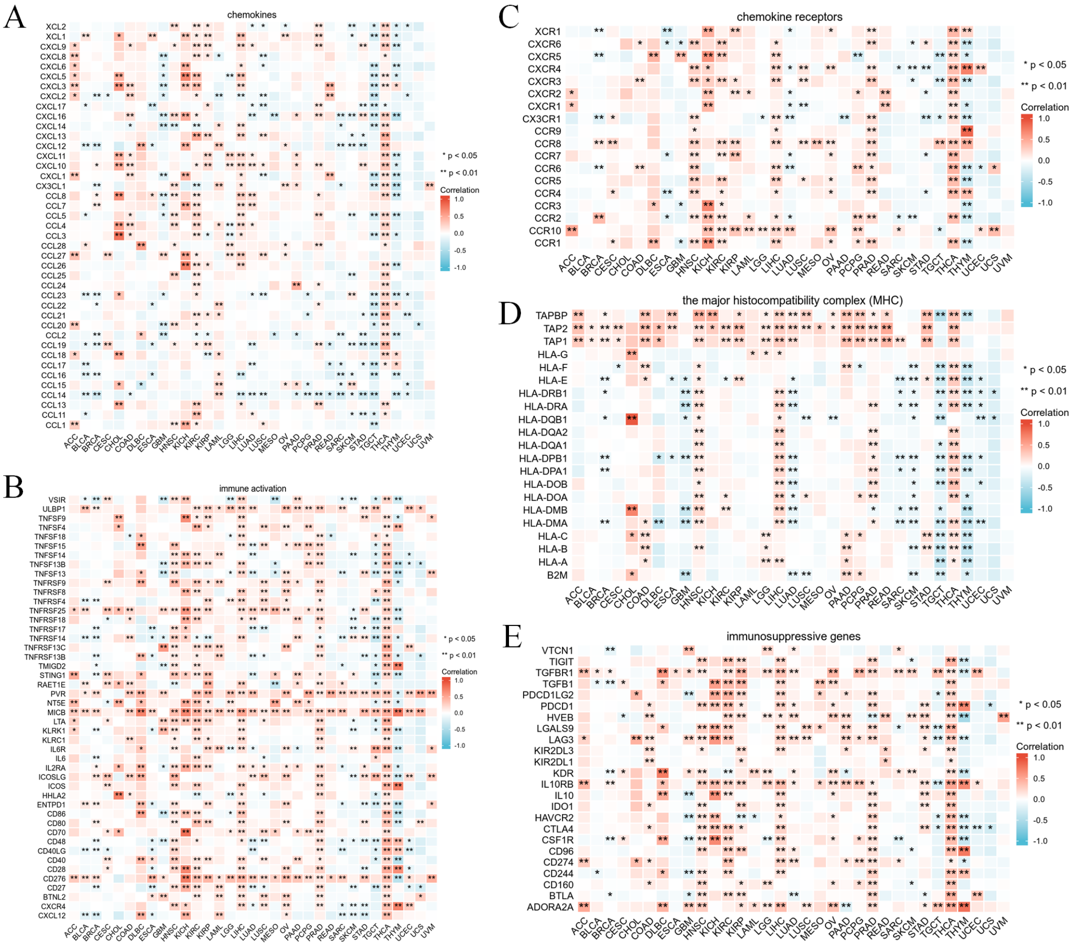

Figure S6. Co-expression of CCNF and immune-related genes.

(A) Chemokines. (B) Immune activation. (C) Chemokine receptors. (D) MHC genes. (E) Immunosuppressive genes. Red represents positive correlation, blue represents negative correlation, and the darker the color, the stronger the correlation. *$p < 0.05$, **$p < 0.01$.

Figure S7

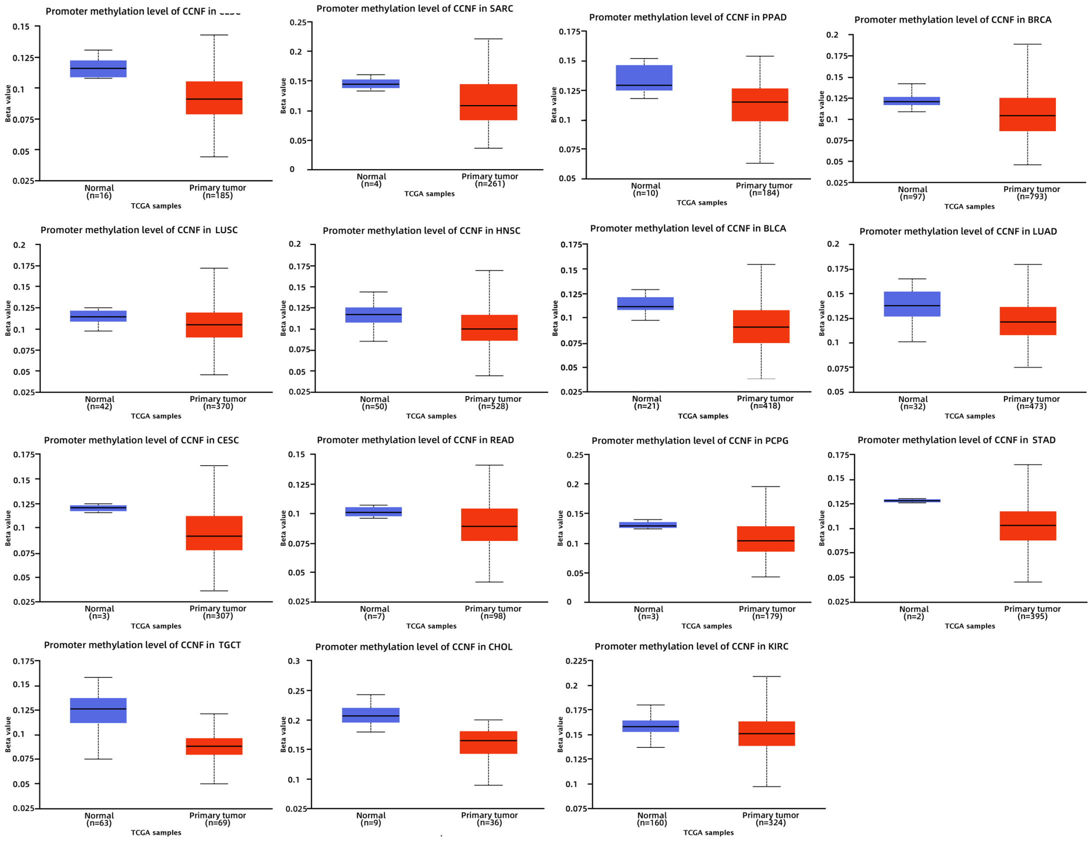

Figure S7. The promoter methylation level of CCNF in cancers.

A different beta value cutoff has been considered to indicate hypomethylation (beta-value < 0.25).

Figure S8

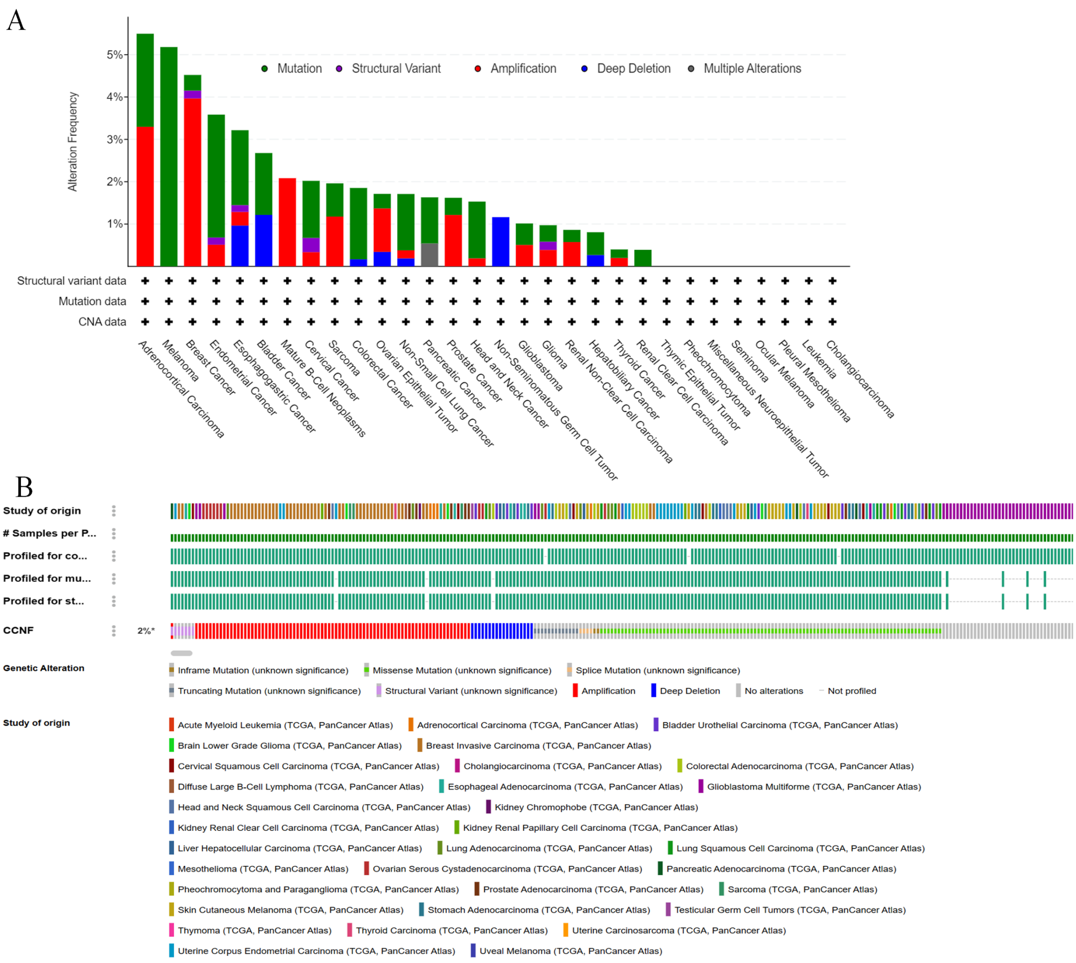

Figure S8. Mutation of CCNF.

(A) Alteration frequency of CCNF. (B) OncoPrint visual summary of alterations in a query of CCNF from cBioPortal.

Figure S9

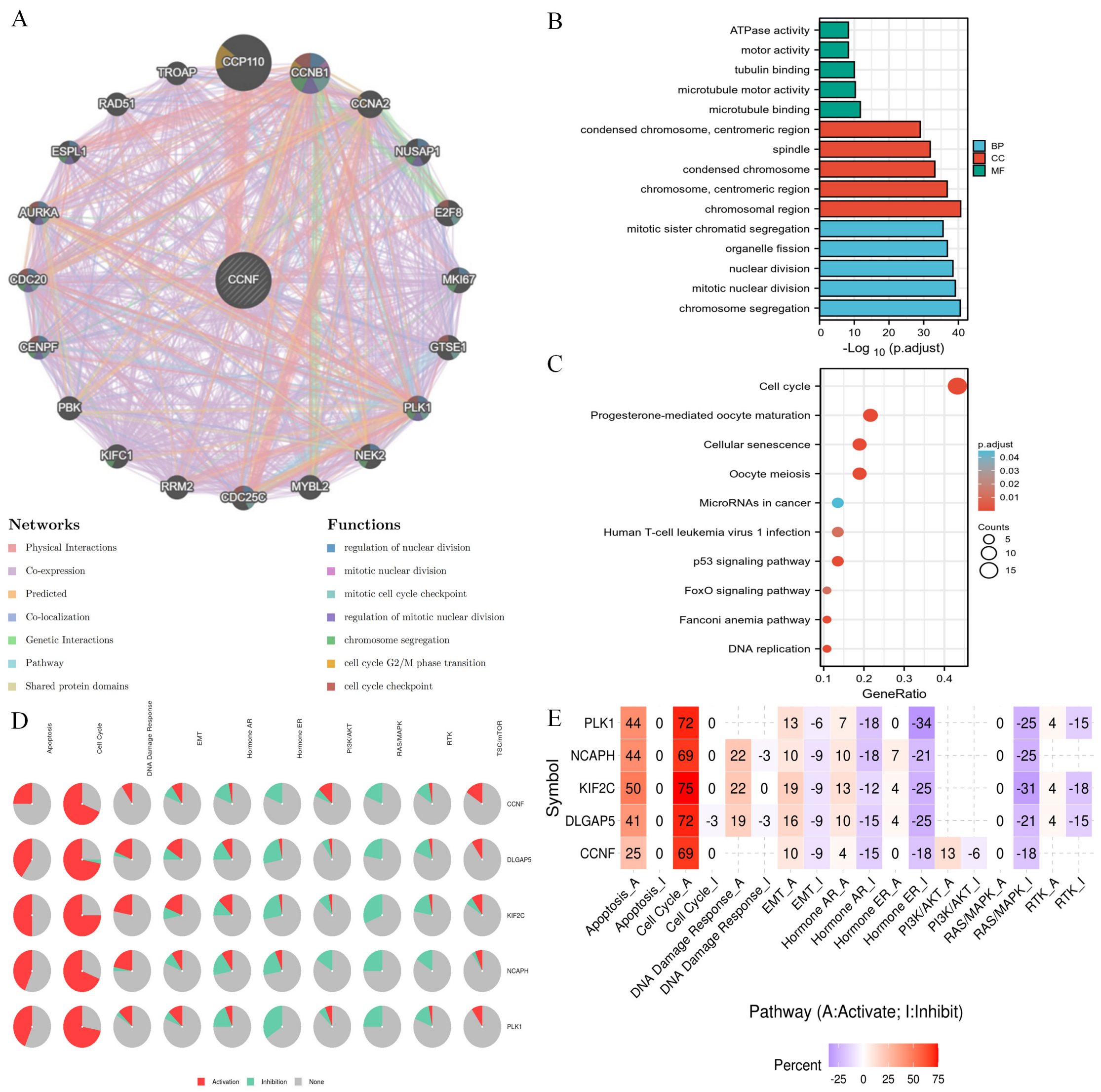

Figure S9. Functional annotation of CCNF in pan-cancer.

(A) The gene–gene interaction network of CCNF from GeneMANIA. (B) The GO pathway analysis based on CCNF binding and interacting genes. (C) The KEGG pathway analysis based on CCNF binding and interacting genes. (D) The pathway activity module of GSCALite was used to investigate the impact of the gene set (CCNF、PLK1、KIF2C、DLGAP5、NCAPH) on tumor pathway activity in pan-cancer. (E) The percentage of cancers in which mRNA expression of the gene set had a potential impact on pathway activity.

Supplementary Table 1.

Table S1.Top 100 genes similar to CCNF from GEPIA2 database

Gene Symbol	Gene ID	PCC
PLK1	ENSG00000166851.14	0.77
KIF2C	ENSG00000142945.12	0.76
DLGAP5	ENSG00000126787.12	0.75
NCAPH	ENSG00000121152.9	0.75
ESPL1	ENSG00000135476.11	0.75
KIF11	ENSG00000138160.5	0.74
RACGAP1	ENSG00000161800.12	0.73
BUB1	ENSG00000169679.14	0.73
CDCA5	ENSG00000146670.9	0.73
GTSE1	ENSG00000075218.18	0.73
CCNB2	ENSG00000157456.7	0.73
KIFC1	ENSG00000237649.7	0.73
NCAPG	ENSG00000109805.9	0.73
LMNB1	ENSG00000113368.11	0.73
TIMELESS	ENSG00000111602.11	0.72
CCNA2	ENSG00000145386.9	0.72
HJURP	ENSG00000123485.11	0.72
RAD51	ENSG00000051180.16	0.72
RCC2	ENSG00000179051.13	0.72
EXO1	ENSG00000174371.16	0.72
KIF4A	ENSG00000090889.11	0.72
TPX2	ENSG00000088325.15	0.72
NUSAP1	ENSG00000137804.12	0.71
CENPA	ENSG00000115163.14	0.71
PLK4	ENSG00000142731.10	0.71
KIF20A	ENSG00000112984.11	0.71
KIF23	ENSG00000137807.13	0.71
ZWINT	ENSG00000122952.16	0.71
CCNB1	ENSG00000134057.14	0.71
MKI67	ENSG00000148773.12	0.7
CDC25A	ENSG00000164045.11	0.7
C16orf59	ENSG00000162062.14	0.7
TTK	ENSG00000112742.9	0.7
ERCC6L	ENSG00000186871.6	0.7
KIF14	ENSG00000118193.11	0.7
CDCA3	ENSG00000111665.11	0.7
GSG2	ENSG00000177602.5	0.7
OIP5	ENSG00000104147.8	0.7

DTL	ENSG00000143476.17	0.69
CENPF	ENSG00000117724.12	0.69
KPNA2	ENSG00000182481.8	0.69
MCM6	ENSG00000076003.4	0.69
SGOL1	ENSG00000129810.14	0.69
TICRR	ENSG00000140534.13	0.69
ORC1	ENSG00000085840.12	0.69
KIF18B	ENSG00000186185.13	0.68
GINS1	ENSG00000101003.9	0.68
AUNIP	ENSG00000127423.10	0.68
SKA1	ENSG00000154839.9	0.68
FANCI	ENSG00000140525.17	0.68
INCENP	ENSG00000149503.12	0.68
CDC25C	ENSG00000158402.18	0.68
RRM2	ENSG00000171848.13	0.67
MCM4	ENSG00000104738.16	0.67
FOXM1	ENSG00000111206.12	0.67
KIAA1524	ENSG00000163507.13	0.67
ASPM	ENSG00000066279.16	0.67
MCM10	ENSG00000065328.16	0.67
AURKB	ENSG00000178999.12	0.67
CENPO	ENSG00000138092.10	0.67
NCAPG2	ENSG00000146918.19	0.67
CHEK1	ENSG00000149554.12	0.67
CDT1	ENSG00000167513.8	0.67
DAZAP1	ENSG00000071626.16	0.67
RAD54L	ENSG00000085999.11	0.67
ARHGAP11A	ENSG00000198826.10	0.66
PRC1	ENSG00000198901.13	0.66
ORC6	ENSG00000091651.8	0.66
CDCA8	ENSG00000134690.10	0.66
NEK2	ENSG00000117650.12	0.66
TMPO	ENSG00000120802.13	0.66
FEN1	ENSG00000168496.3	0.66
STIL	ENSG00000123473.15	0.66
FANCD2	ENSG00000144554.10	0.66
CENPI	ENSG00000102384.13	0.66
PTBP1	ENSG00000011304.16	0.66
KIF15	ENSG00000163808.16	0.66
RNPS1	ENSG00000205937.11	0.66
MIS18A	ENSG00000159055.3	0.66
ZWILCH	ENSG00000174442.11	0.66
CLSPN	ENSG00000092853.13	0.66
SRSF7	ENSG00000115875.18	0.66

RFWD3	ENSG00000168411.13	0.66
C17orf53	ENSG00000125319.14	0.65
TDP1	ENSG00000042088.13	0.65
WDR62	ENSG00000075702.16	0.65
AURKA	ENSG00000087586.17	0.65
BUB1B	ENSG00000156970.12	0.65
MCM2	ENSG00000073111.13	0.65
CKAP2L	ENSG00000169607.12	0.65
SPC25	ENSG00000152253.8	0.65
KIF18A	ENSG00000121621.6	0.65
EZH2	ENSG00000106462.10	0.65
UBE2T	ENSG00000077152.9	0.65
TROAP	ENSG00000135451.12	0.65
NCAPD2	ENSG00000010292.12	0.65
MYBL2	ENSG00000101057.15	0.65
CENPE	ENSG00000138778.11	0.65
SKA3	ENSG00000165480.15	0.64
CHAF1B	ENSG00000159259.7	0.64

Table S2.Interacting chemicals of CCNF from CTD.

Chemical Name	Chemical ID	Interaction Actions	Chemical Name	Chemical ID	Interaction Actions
1,2,5,6-dibenzanthracene	C026486	increases expression	dorsomorphin	C516138	decreases expression
1,2-Dimethylhydrazine	D019813	increases expression	Dust	D004391	decreases expression
1,4-bis(2-(3,5-dichloropyridyloxy))benzene	C028474	decreases expression	Endosulfan	D004726	increases expression
1-Methyl-4-phenyl-1,2,3,6-tetrahydropyridine	D015632	increases expression	epigallocatechin gallate	C045651	decreases expression
2,2',3',4,4',5-hexachlorobiphenyl	C029790	increases expression	erucylphospho-N,N,N-trimethylpropylammonium	C472787	decreases expression
2,2',4,4'-tetrabromodiphenyl ether	C511295	affects expression	Ethinyl Estradiol	D004997	increases expression
2,3-bis(3'-hydroxybenzyl)butyrolactone	C029497	increases expression	Fenofibrate	D011345	increases expression
2,4,4'-trichlorobiphenyl	C081766	increases expression	Fenretinide	D017313	increases expression
2,4,5,2',4',5'-hexachlorobiphenyl	C014024	increases expression	Formaldehyde	D005557	decreases expression
2,4,5,2',5'-pentachlorobiphenyl	C009828	increases expression	fullerene C60	C069837	increases expression
2,5,2',5'-tetrachlorobiphenyl	C009407	increases expression	furan	C039281	increases expression
2-methyl-4-isothiazolin-3-one	C011506	decreases expression	hydroquinone	C031927	decreases expression
3-((6-(2-methoxyphenyl)pyrimidin-4-yl)amino)phenyl)methane sulfonamide	C000590771	decreases expression	ICG 001	C492448	decreases expression
4-(4-((5-(4,5-dimethyl-2-nitrophenyl)-2-furanyl)methylene)-4,5-dihydro-3-methyl-5-oxo-1H-pyrazol-1-yl)benzoic acid	C584509	decreases expression	incobotulinumtoxinA	C545476	decreases expression
4-(5-benzo(1,3)dioxol-5-yl-4-pyridin-2-yl-1H-imidazol-2-yl)benzamide	C459179	decreases expression	indole-3-carbinol	C016517	affects expression
4-hydroxy-2-nonenal	C027576	decreases expression	Indomethacin	D007213	decreases expression
7-(benzylamino)-1,3,4,8-tetrahydropyrrolo(4,3,2-de)quinolin-8(1H)-one	C553817	decreases expression	Ionomycin	D015759	increases expression
7,8-Dihydro-7,8-dihydroxybenzo(a)pyrene 9,10-oxide	D015123	decreases expression	Irinotecan	D000077146	decreases expression
acetamide	C030686	increases expression	jinfukang	C544151	increases expression
Acetaminophen	D000082	affects expression	Leflunomide	D000077339	decreases expression
Air Pollutants	D000393	increases expression	Lidocaine	D008012	affects expression
Ammonium Chloride	D000643	affects expression	Metformin	D008687	decreases expression
amphotericin B, deoxycholate	C059765	increases expression	Mustard Gas	D009151	decreases expression

drug combination

Arsenic Trioxide	D000077237	decreases expression	Nanotubes, Carbon	D037742	increases expression
AZM551248	C547126	increases expression	n-butoxyethanol	C017096	increases expression
Benzene	D001554	increases expression	nickel chloride	C022838	affects expression
Benzo(a)pyrene	D001564	decreases expression	nickel monoxide	C028007	increases expression
benzo(b)fluoranthene	C006703	increases expression	Niclosamide	D009534	decreases expression
beta-methylcholine	C044887	affects expression	NSC 689534	C558013	decreases expression
bisphenol A	C006780	decreases expression	NSC668394	C570897	decreases expression
Bortezomib	D000069286	decreases expression	Oxaliplatin	D000077150	decreases expression
Butylated Hydroxytoluene	D002084	increases expression	Oxazolone	D010081	increases expression
butyraldehyde	C018475	decreases expression	Oxygen	D010100	decreases expression
Cadmium	D002104	decreases expression	Ozone	D010126	increases expression
Caffeine	D002110	increases expression	Paclitaxel	D017239	decreases expression
Calcitriol	D002117	decreases expression	palbociclib	C500026	decreases expression
Cannabidiol	D002185	decreases expression	PCB 180	C410127	increases expression
Carbamazepine	D002220	affects expression	pentabromodiphenyl ether	C086401	decreases expression
Carbon Tetrachloride	D002251	affects expression	perfluoro-n-nonanoic acid	C101816	decreases expression
Celecoxib	D000068579	decreases expression	phenethyl isothiocyanate	C058305	decreases expression
Chlorpromazine	D002746	decreases expression	Phenobarbital	D010634	affects expression
chromium hexavalent ion	C074702	affects expression	Phenylmercuric Acetate	D010662	decreases expression
Cisplatin	D002945	decreases expression	pirinixic acid	C006253	increases expression
Clofibrate	D002994	increases expression	Polychlorinated Biphenyls	D011078	affects expression
cobaltous chloride	C018021	decreases expression	polyhexamethyleneguanidine	C060540	affects expression
Copper	D003300	decreases expression	potassium chromate(VI)	C027373	decreases expression
Copper Sulfate	D019327	decreases expression	Pregnenolone Carbonitrile	D011285	decreases expression
Coumestrol	D003375	increases expression	Progesterone	D011374	decreases expression
cyanoginosin LR	C057862	decreases expression	propionaldehyde	C005556	decreases expression
Cyclosporine	D016572	decreases expression	Propylthiouracil	D011441	increases expression
Decitabine	D000077209	decreases expression	Quercetin	D011794	decreases expression
Diazinon	D003976	affects expression	Resveratrol	D000077185	decreases expression
Dibutyl Phthalate	D003993	decreases expression	Rotenone	D012402	decreases expression
Diclofenac	D004008	affects expression	S-(1,1,2,2-tetrafluoroethyl)cystein e	C064116	decreases expression
dicrotophos	C000944	increases expression	seocalcitol	C078903	decreases expression
Dieldrin	D004026	affects expression	Silicon Dioxide	D012822	increases expression
Dietary Fats	D004041	increases expression	Silver	D012834	decreases expression
Diethylnitrosamine	D004052	increases expression	Smoke	D012906	decreases expression
Dinitrochlorobenzene	D004137	increases expression	sodium arsenite	C017947	decreases expression
Tobacco Smoke Pollution	D014028	decreases expression	Soman	D012999	increases expression
Toluene 2,4-Diisocyanate	D014051	increases expression	Soot	D053260	increases expression
Topotecan	D019772	decreases expression	Sunitinib	D000077210	decreases expression
Tretinoin	D014212	decreases expression	tamibarotene	C061133	decreases expression
Trichloroethylene	D014241	decreases expression	Temozolomide	D000077204	increases expression

trimellitic anhydride	C015559	increases expression	tert-Butylhydroperoxide	D020122	increases expression
tris(1,3-dichloro-2-propyl)phosphate	C016805	decreases expression	Testosterone	D013739	decreases expression
Troglitazone	D000077288	decreases expression	Tetrachlorodibenzodioxin	D013749	affects expression
Tungsten	D014414	increases expression	Tetradecanoylphorbol Acetate	D013755	increases expression
Tunicamycin	D014415	decreases expression	Thapsigargin	D019284	decreases expression
Valproic Acid	D014635	affects expression	Thioacetamide	D013853	decreases expression
vanadyl sulfate	C034028	decreases expression	Vitallium	D014800	decreases expression

Table S3. Analysis of the relationship between CCNF and genes in the CTD database.

Gene	Similarity Index	Common Interacting Chemicals
FBXO5	0.415	83
DLGAP5	0.408256881	89
CDCA2	0.396984925	79
NUSAP1	0.39380531	89
CDCA3	0.393364929	83
UBE2T	0.385786802	76
ASF1B	0.385	77
ASPM	0.382882883	85
CENPA	0.377990431	79
KIFC1	0.371980676	77
TIMELESS	0.370967742	69
CDT1	0.370192308	77
KIF20A	0.369294606	89
PRIM1	0.368159204	74
TK1	0.367816092	96
PLK1	0.366412214	96
CENPF	0.363265306	89
CDCA8	0.360189573	76
BUB1B	0.358649789	85
NCAPG	0.358585859	71
